# Meteorological Gbservations for September, 1857, at Atlanta, Ga.

**Published:** 1857-10

**Authors:** J. G. Westmoreland


### METEOROLOGICAL OBSERVATIONS FOR SEPTEMBER, 1857, AT ATLANTA, GA.

| SEPTEMBER. | THERMOMETER. | | | BAROMETER. | | | WIND. | REMARKS. |
|---|---|---|---|---|---|---|---|---|
| | 7 A. M. | 2 P. M. | 7 P. M. | 7 A. M. | 2 P. M. | 7 P. M. | | |
| 1 | 66 | 80 | 74 | 30.20 | 30.30 | 30.25 | E. | Fair. |
| 2 | 70 | 76 | 68 | 30.25 | 30.30 | 30.20 | E. | Fair. |
| 3 | 64 | 76 | 72 | 30.22 | 30.22 | 30.25 | N. E. | Fair. |
| 4 | 72 | 80 | 76 | 30.30 | 30.30 | 30.25 | E. | Fair. |
| 5 | 76 | 88 | 80 | 30.22 | 30.30 | 30.22 | E. | Hazy. |
| 6 | 68 | 86 | 84 | 30.20 | 30.20 | 30.15 | E. | Fair. |
| 7 | 72 | 86 | 70 | 30.27 | 30.20 | 30.20 | E. | Fair. |
| 8 | 70 | 76 | 72 | 30.25 | 30.32 | 30.30 | E. | Fair. |
| 9 | 72 | 76 | 76 | 30.30 | 30.27 | 30.20 | E. | Fair. |
| 10 | 58 | 80 | 74 | 30.15 | 30.20 | 30.12 | E. | Fair. |
| 11 | 72 | 80 | 70 | 30.20 | 30.15 | 30.07 | E. | Fair. |
| 12 | 62 | 88 | 78 | 30. | 30.07 | 30. | E. | Fair. |
| 13 | 64 | 88 | 86 | 30. | 30.05 | 30.02 | E. | Fair. |
| 14 | 68 | 88 | 86 | 30.10 | 30.12 | 30.15 | E. | Fair. |
| 15 | 78 | 90 | 82 | 30.15 | 30.15 | 30.12 | W. | Fair. |
| 16 | 80 | 94 | 84 | 30.12 | 30.20 | 30.15 | S. W. | Fair. |
| 17 | 76 | 92 | 80 | 30.06 | 30.20 | 30.12 | E. | Fair. |
| 18 | 70 | 80 | 80 | 30.10 | 30.12 | 30.06 | S. W. | Hazy. |
| 19 | 72 | 80 | 96 | 30. | 30. | 30. | S. | Cloudy—Rain ⅛. |
| 20 | 72 | 82 | 68 | 30. | 30. | 29.95 | W. | Fair. |
| 21 | 58 | 82 | 74 | 29.95 | 30.05 | 27.97 | N. W. | Hazy. |
| 22 | 58 | 76 | 60 | 29.95 | 29.95 | 29.90 | E. | Cloudy. |
| 23 | 56 | 70 | 58 | 29.95 | 29.92 | 29.90 | N W. | Fair. |
| 24 | 52 | 76 | 70 | 30. | 30. | 30. | N. W. | Fair. |
| 25 | 52 | 70 | 70 | 30. | 30.05 | 30.10 | N W. | Fair. |
| 26 | 70 | 80 | 70 | 30.30 | 30.25 | 30.20 | W. | Fair. |
| 27 | 58 | 78 | 76 | 30.20 | 30.25 | 30.25 | W. | Fair. |
| 28 | 80 | 86 | 70 | 30.35 | 30.30 | 30.25 | W. | Fair. |
| 29 | 60 | 70 | 60 | 30.25 | 30.20 | 30.25 | N. | Fair. |
| 30 | 42 | 70 | 62 | 30.20 | 30.25 | 30.20 | E. | Fair. |

Furnished by

J. G. WESTMORELAND, M. D.

